# Rapid-progressing progressive multifocal leukoencephalopathy in two patients newly diagnosed with HIV: case series and review of literature

**DOI:** 10.1007/s13365-023-01115-5

**Published:** 2023-02-11

**Authors:** Barbara Badura, Szymon Barczak, Tomasz Mikuła, Alicja Wiercińska-Drapało

**Affiliations:** 1 Students’ Scientific Society of the Department of Infectious and Tropical Diseases and Hepatology, Medical University of Warsaw, Warsaw, Poland; 2 Department of Infectious and Tropical Diseases and Hepatology, Medical University of Warsaw, Warsaw, 02-091 Poland; 3 Warsaw’s Hospital for Infectious Diseases, Warsaw, 01-201 Poland

**Keywords:** HIV, AIDS, PML, Focal neurologic signs, JCPyV, Rapid progression

## Abstract

The JC Polyomavirus (JCPyV) is a virus of global distribution and is usually kept under control by the immune system. In patients with AIDS, a latent JCPyV infection can reactivate and develop into progressive multifocal leukoencephalopathy (PML). Around half of the patients with PML die within 2 years since the diagnosis, yet in rare cases, the disease advances significantly quicker and seems to be insusceptible to any medical actions. In our clinic, we observed two cases of such course in HIV-positive patients in the AIDS stage. On admission, both patients had mild neurological symptoms such as dizziness, vision disturbances, and muscle weakness. Both had extremely low CD4 lymphocyte count (7 cells/μL, 40 cells/μL) and high HIV-1 viral load (VL) (50,324 copies/ml, 78,334 copies/ml). PML was confirmed by PCR for JCPyV DNA in cerebrospinal fluid (CSF) coupled with clinical and radiological features. Despite receiving though antiretroviral (ARV) treatment paired with intra-venous (IV) steroids, the disease progressed rapidly with neurological manifestations exacerbating throughout the few weeks following the admission. Eventually, both patients developed respiratory failure and died within less than 3 months after the onset of the neurological symptoms. Even though such curse of the disease is not common, it should be a warning to all how deadly both PML and AIDS can be and remind doctors to offer testing even to asymptomatic patients.

## Introduction

In 2021, around 38 million people were living with human immunodeficiency virus (HIV) in the world, and around 1.5 million new infections were observed that year (Fact sheet - Latest global and regional statistics on the status of the AIDS epidemic. | UNAIDS. (n.d.) https://www.unaids.org/en/resources/documents/2022/UNAIDS_FactSheet. Accessed 15 November 2022). More than half (54%) of newly diagnosed patients belong to the group of late-presenters (45% in Poland) (Jabłonowska et al. 2021; Raffetti et al. 2016). Late presentation of an HIV infection is defined as a diagnosis of HIV with a CD4 lymphocyte count < 350 cells/μL, or the occurrence of an acquired immunodeficiency syndrome (AIDS)-defining event, regardless of the CD4 lymphocyte count (Wójcik-Cichy et al. 2018). Such a state is associated with several adverse outcomes including an increased risk of clinical progression, blunted immune recovery on highly active antiretroviral therapy, and a greater risk of drug toxicity (Waters and Sabin 2011). Due to the low CD4 lymphocyte count, late presenters are more at risk of developing opportunistic infections, some of which involve the central nervous system (CNS) and can cause neurological symptoms. An example of such infection is progressive multifocal leukoencephalopathy (PML) caused by the Polyomavirus JC (JCPyV).

Primary JCPyV infection usually occurs in childhood and remains asymptomatic; thus, most of the infected become chronic carriers. In those patients, the latent infection can be reactivate and develop into PML if they reach a state of decreased immunity (Guidelines for the Prevention and Treatment of Opportunistic Infections in Adults and Adolescents with HIV How to cite the adult and adolescent opportunistic infection guidelines: panel on guidelines for the prevention and treatment of opportunistic infections in adults and adolescents with HIV. Guidelines for the Prevention and Treatment of Opportunistic Infections in Adults and Adolescents. (n.d.). https://clinicalinfo.hiv.gov/en/. Accessed 15 November 2022).

During the course of PML, the virus destroys oligodendrocytes and their myelin processes (Snopková et al. 2019). The demyelinating lesions can involve different regions of the brain; thus, specific neurological deficits vary from location—aphasia, hemiparesis, and hemisensory deficits, and hemianopsia to cerebellar dysmetria and ataxia, and usually leads to death (Guidelines for the Prevention and Treatment of Opportunistic Infections in Adults and Adolescents with HIV How to cite the adult and adolescent opportunistic infection guidelines: panel on guidelines for the prevention and treatment of opportunistic infections in adults and adolescents with HIV. Guidelines for the Prevention and Treatment of Opportunistic Infections in Adults and Adolescents. (n.d.). https://clinicalinfo.hiv.gov/en/. Accessed 15 November 2022).

There are two pathways used for diagnosing PML proposed by the American Academy of Neurology (AAN). One of the two is based on histopathological evidence (demyelination, bizarre astrocytes, and enlarged oligodendroglial nuclei) paired with the techniques to reveal the presence of JCPyV, whereas the second one requires the presence of clinical and radiological features consistent with the diagnosis, together with the detection of JCPyV DNA in cerebrospinal fluid (CSF) (Berger et al. 2013).

Currently, there is no specific PML treatment available. Reconstituting the protective immunity or reversing the immunosuppression is so far the best way to eliminate JCPyV infection from the CNS and to overcome PML (Snopková et al. 2019). However, in many cases, those targets cannot be achieved; therefore, new advanced treatment options are being explored. A noteworthy example is the use of immune checkpoint inhibitors pembrolizumab or nivolumab, both of which are monoclonal antibodies that target programmed cell death protein-1 (PD-1) (Koralnik 2019).

Even though antiretroviral treatment (ARV) is widely accessible, the mortality rate in patients with HIV-associated PML is up to 30% after 1 year and 50–60% after 2 years after the diagnosis (Kartau et al. 2019). Rarely, the disease can progress very fast and lead to death within just weeks despite treatment being conducted.

We analyzed the clinical course of two HIV-infected patients with PML because of rapidly progressing infections.

## Case series

### Patient A

Since the end of July 2020, a 27-year-old male with no significant medical history reported balance and gait disturbances due to dizziness and muscle weakness in the left upper limb. Moreover, ptosis and visual field loss on the left side were observed. In August 2020, he was hospitalized in the Department of Neurology. The neurological examination showed nystagmus, spastic paresis, and positive Babinski’s sign on the left side. Magnetic resonance imaging (MRI) of the head presented subcortical white matter swelling in the regions of the right occipital, temporal, and parietal lobes. Since a new diagnosis of an HIV infection was made, the patient was transferred to the Department of Infectious and Tropical Diseases and Hepatology for further diagnosis and treatment.

At the time of admission, laboratory tests showed decreased immunological results (Table 1). In performed tests, IgG and IgM antibodies against *Toxoplasma gondii* and cytomegalovirus (CMV) were not detected; the Venereal Disease Research Laboratory (VDRL) test was also negative. HIV infection was confirmed by Western blot test and PCR HIV-1 RNA blood viral load (VL).

**Table 1:** The baseline immunological and virological data of patients A and B during observation

	**Patient A**		**Patient B**	
	**On admission**	**After 4 weeks**	**On admission**	**After 6 weeks**
CD4 lymphocyte count (cells/μL)	7	6	40	98
CD8 lymphocyte count (cells/μL)	238	154	602	1,191
CD4/CD8 ratio	0.03	0.04	0.07	0.08
HIV-1 VL (copies/ml)	50,324	not detected	78,334	205
JCPyV DNA copies in CSF (copies/ml)	113,000,000	sample not taken	396	Sample not taken

A confirmatory PCR result for JCPyV DNA in CSF was obtained making the diagnosis of PML certain. The patient’s condition and neurological symptoms aggravated, and he was no longer in contact. A percutaneous endoscopic gastrostomy (PEG) tube was inserted and oxygen therapy started.

**Fig. 1:**
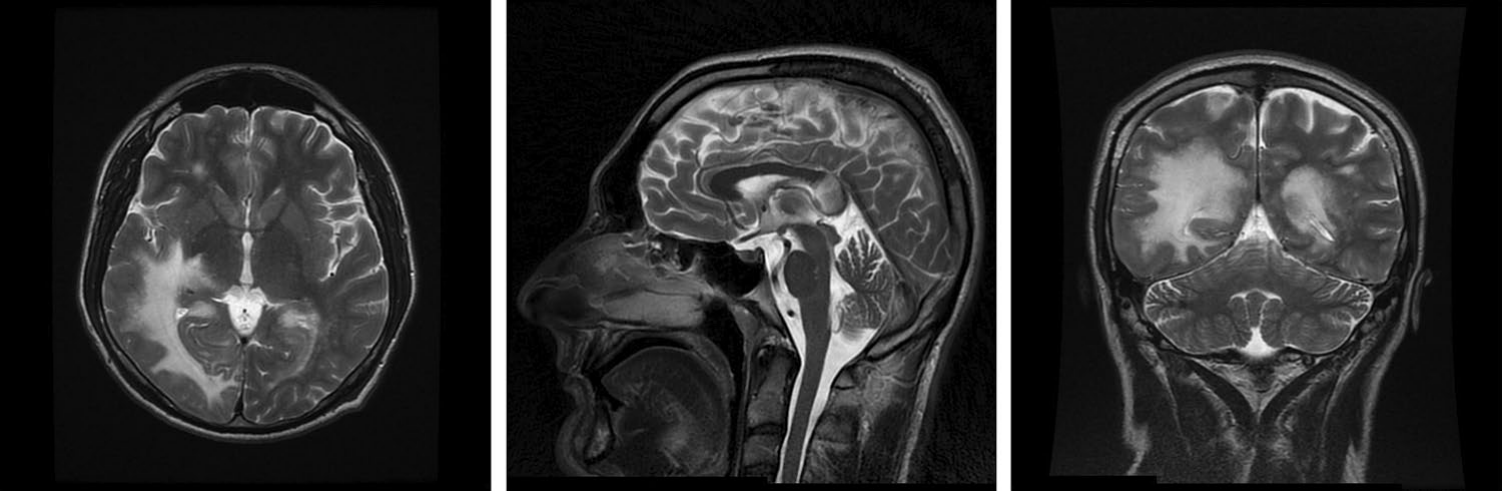
Patient A: T2-weighted brain MRI, transverse, sagittal, and coronal planes

The single-photon emission computed tomography (SPECT) MRI of the CNS showed focal lesions of an unknown etiology (Fig. 1); both PML and lymphoma were suspected. The patient was started on ARV—a combination of bictegravir, emtricitabine, and tenofovir alafenamide (BIC/FTC/TAF), along with dexamethasone and mannitol. However, evidence of immune restoration was never observed.

Another MRI of the brain presented a slight progression of the lesions, not enhancing contrast. A lumbar puncture was performed to examine the CSF for lymphoma cells and signs of opportunistic infections. The patient was consulted by a neurosurgeon since performing a stereotactic brain biopsy was considered.

A seizure attack accompanied by a blood saturation decrease to 87% occurred. Stertorous tachypnoea and dilated pupils with fever were present. A blood sample was taken for the culture of aerobic and anaerobic bacteria. Sodium valproate, diazepam, and paracetamol were administered. The patient was in severe condition and feverish. In the following days, the respiratory failure progressed due to pneumonia confirmed by chest X-ray. A decision was made to switch ceftriaxone for meropenem.

Accumulating purulent mucus in the respiratory tract caused dyspnea. A new MRI of the brain showed progression in the white matter of both hemispheres and the cerebellum. During the following days, the patient’s condition deteriorated, and he developed a pulmonary infection and respiratory failure. Blood cultures were positive for multi-drug resistant pathogens: *Candida albicans* and *Acinetobacter baumannii*. Despite the applied treatment, no clinical improvement was achieved.

The patient died in October 2020, approximately 11 weeks after the onset of the neurological symptoms.

### Patient B

In March 2019, a 38-year-old female, with no significant medical history, started complaining of neurological symptoms: dizziness, blurred vision, diplopia, and muscle weakness on the left side during hospitalization in the department of rheumatology. Neurological examination showed positive Babinski’s sign on the right side, ataxia of the left limbs, and nystagmus. A screening test for HIV was performed, and its positive result was confirmed by western blot. A previous test for HIV in 2010 was negative.

**Fig. 2:**
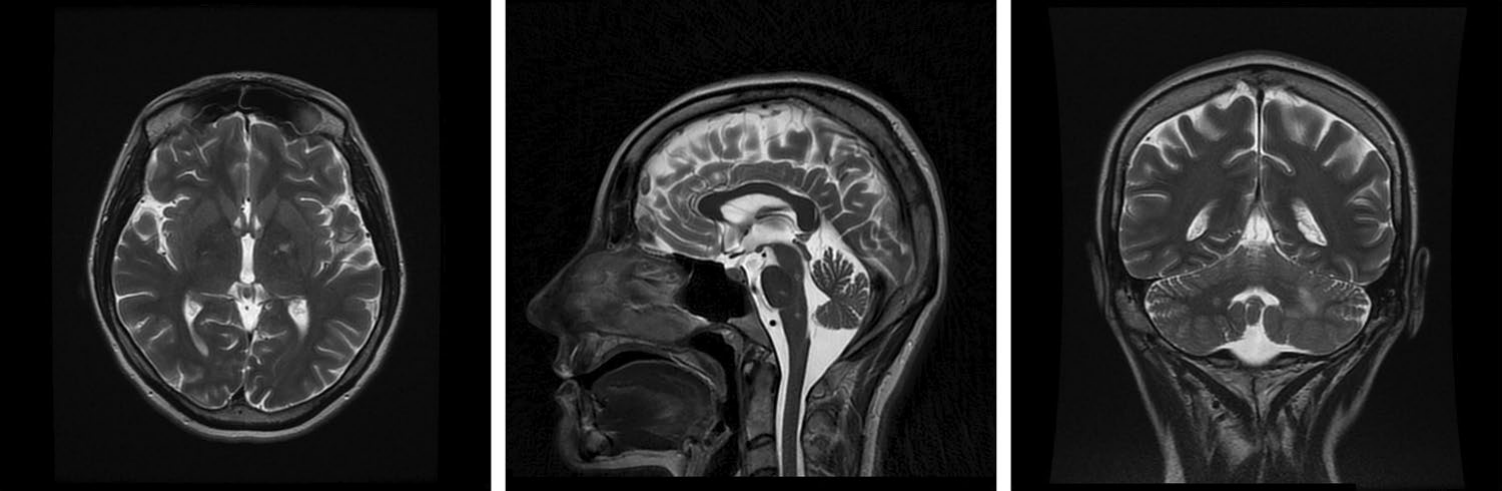
Patient B: T2-weighted brain MRI, transverse, sagittal, and coronal planes

Later that month, she was admitted to the Department of Infectious and Tropical Diseases and Hepatology with the baseline immunological and virological data as presented in Table 1. The patient was started on ARV—tenofovir alafenamide, emtricitabine, and darunavir with cobicistat (TAF/FTC/DRV/c), along with cotrimoxazole as *Pneumocystis* pneumonia prophylaxis. Furthermore, a lumbar puncture was performed, and the CSF was cultured for bacteria, fungi, and *Mycobacterium tuberculosis* and sent for PCR for JCPyV DNA. MRI showed lesions in the left cerebellar peduncle, on the left side of the pons, showing no enhancement on contrast and with increased signal in diffusion-weighted imaging (DWI) (Fig. 2). The patient was started on anti-toxoplasmosis treatment consisting of pyrimethamine with sulfadiazine due to the presence of IgG antibodies against *Toxoplasma gondii*. Her status was stable. At that time, she requested to be discharged and left the hospital without any additional symptoms apart from dysphagia.

Five days later, she was admitted once again because her overall condition worsened. The patient was complaining of headaches, nausea, vomiting, and increased balance and gait impairment. Additional neurological findings such as central paresis of the left facial nerve and ataxia of the right limbs started to appear. Dexamethasone was administered. Her following MRIs showed progression of the lesions in the cerebellum without signs of hydrocephalus. Toxoplasmosis was excluded, and its treatment was terminated. Since the PCR for the JCPyV DNA in CSF result was not yet available, both lymphoma and PML were suspected. Even though the patient presented signs of initiatory immune restoration, the neurological symptoms strongly aggravated during the hospitalization, everyday activities started to cause difficulties, and slurred speech appeared, yet her cognitive functions were not affected. The patient was discharged.

After 4 days, the patient was admitted again complaining of symptoms from the respiratory tract. Due to severe dysphagia, the patient was offered an insertion of a PEG tube, yet refused and demanded to be discharged. On the next day, she was admitted again in critical condition. At this moment, confirmatory PCR results for the JCPyV DNA in CSF were obtained. Day by day, the patient’s condition was deteriorating, mucus cumulating in the upper respiratory tract caused dyspnea, she required to be fed parenterally and stopped passing stools, and eventually was responsive only to pain stimuli. Because of the trismus, it got more difficult to remove the purulent mucus, and oxygen therapy was necessary. During all that time, the patient’s body temperature remained within the normal range. Due to the lack of other accessible treatment options, the patient was administered morphine, and it was decided not to resuscitate in case of a cardiac arrest. She died in May 2019, approximately 10 weeks after the onset of the neurological symptoms.

For more baseline immunological data, see Table 1.

## Discussion

Reactivation of a latent JCPyV infection and its development into PML generally requires the immune system to be compromised—the severity of such a state can be measured with several diagnostic methods, CD4 lymphocyte count being the most common in HIV-positive patients. Available data states that a CD4 lymphocyte count of < 200 cells/µl and a considerable reduction in CD4/CD8 ratio are risk factors of PML in people living with HIV (Sokol et al. 2017). Both of our patients met this criterion and thus had immunological predispositions for the reactivation of the JCPyV infection.

In the criteria for the diagnosis of PML suggested by AAN, histopathological features serve as one of two possible approaches. The other one is based on radiographic and clinical features coupled with the presence of the JCPyV DNA in the CSF (Berger et al. 2013). Neither of the featured patients underwent a brain biopsy since it was decided that they would not benefit from the procedure. In order not to expose the patients to any unnecessary trauma and bearing in mind their already advanced clinical condition, we chose to follow the latter of the two available methods. In consequence, we were able to reach unequivocal diagnoses faster and less intrusively.

Both of the featured patients presented late and reached the hospital at the AIDS stage. The reason for this may be insufficient screening for HIV which could allow earlier detection of the infection, and, as a consequence, adequate medical care could be provided sooner. Unfortunately, only one of the patients had undergone a test for HIV prior to the hospitalization, yet it took place 9 years earlier. According to the recommendations suggested by the Centers for Disease Control and Prevention (CDC), every individual between 13 and 64 years of age should be routinely tested for HIV at least once, while those from the risk groups are advised to be screened at least annually (Screening in Clinical Settings | Screening for HIV | Clinicians | HIV | CDC. (n.d.). https://www.cdc.gov/hiv/clinicians/screening/clinical-settings.html. Accessed 11 January 2023). Despite these recommendations, currently available data shows that fewer than 40% of people in the USA had ever been tested (CDC Press Release: Most Americans Have Never Had an HIV Test, New Data Show | CDC Online Newsroom | CDC. (n.d.). https://www.cdc.gov/media/releases/2019/p0627-americans-hiv-test.html. Accessed 11 January 2023). Wurm et al. (2019) managed to identify barriers to accessing HIV testing services. His paper states that the factor of greatest influence in the decision-making process is the individual risk perception and anxiety regarding stigmatization.

After analyzing articles published from 2010 and 2020, Sun et al. determined factors associated with late presentation of an HIV infection in China. According to his meta-analysis, the most at risk were patients above 50 years of age, married, with heterosexual contact as risk factor for infection and diagnosed in medical institutions. Similarly, a wide study conducted in the Netherlands by De Coul et al. (2016) identified characteristics associated with higher odds of late presentation, the most important being: heterosexual male, injecting drug use, and age above 50. Furthermore, this author demonstrated that the percentage of late presentation decreased over time but only among men having sex with men (MSM) and not among heterosexual patients. Both patients, A and B, were diagnosed in a hospital and had history of heterosexual contacts, and patient B was a married woman. However, neither of them was older than 50, and their history of IV drug use remains uncertain. Therefore, they both had some factors facilitating late presentation.

In both cases, the disease progressed similarly as it would in the times before effective therapy for AIDS. In those days, HIV-associated PML almost always resulted in death within months (Dunham et al. 2020), and only 10% of the patients were alive 1 year after the PML diagnosis (Berger et al. 1998). Nowadays, in the era of widely available antiretroviral drugs, the clinical course of PML is slower. According to Engsig et al., introducing ARV increased median survival time significantly: from 0.4 years to 1.8 years (Engsig et al. 2009). Furthermore, some patients live much longer, Lima et al. (2010) described 24 patients (23 of them being HIV-positive, all receiving ARV) whose survival time exceeded years, and Yoganathan et al. (2012), a patient who was still alive 12 years after the diagnosis. All this data stands in contrast to our patients’ outcomes.

Other authors also discuss cases in which the disease progressed rapidly. De Paula e Silva et al. (2011) presented a case of a 31-year-old HIV-infected male who is similar with our patients died 3 months after the diagnosis and did not respond to the ARV treatment. However, unlike the featured patients, ARV drugs in de Paula e Silva’s patient were administered at a later stage, after performing an MRI which revealed very advanced diffuse lesions in the subcortical white matter typical for PML and not right after detecting the HIV infection. What is also interesting, his patient’s CD4 lymphocyte count was 427 cells/µl on admission, whereas CD4 lymphocyte counts in our patients were low. Despite these differences, the clinical outcome in all three patients was very alike.

Another example of a paced progression of PML was described by Krey et al. (2019) whose paper features a 68-year-old HIV-negative male who developed the disease despite being fully immunocompetent and not receiving any immunosuppressive therapy. The patient presented typically, with mild neurological symptoms which later aggravated and recurring seizures appeared. He was treated with antiepileptic drugs, intravenous corticosteroids and mirtazapine. The patient was discharged with some neurological symptoms still persistent. In the following weeks, he developed severe dysphagia and died of pulmonary failure due to a respiratory infection. Even though this clinical course shares many similarities with our patients, the underlying cause of JCPyV reactivation differs. Therefore, the featured patient was not receiving ARV treatment but mirtazapine which was reported to be beneficial in an immunocompetent patient with PML (Christakis et al. 2013).

A very different disease course was described by Yoganathan et al. (2012). This author presented a case of a 43-year-old male with AIDS who recovered from PML to a great extent. In March 1999, the patient was admitted due to neurological symptoms with a CD4 lymphocyte count of 34 cells/µl and VL 800,789 copies/ml and received ARV treatment. The patient was last reviewed in October 2011. He still presented residual neurological symptoms, yet his immune system fully recovered—his CD4 lymphocyte count was 574 cells/µl, and VL was not detectable. Even though the onset of the disease and immunological risk factors were consistent with what we observed in our patients, the clinical course was significantly more favorable.

The greatest difference observed between the featured patients is their response to the ARV treatment. In both cases, the VL dropped significantly in the control laboratory tests, however, an increase in CD4 and CD8 lymphocyte counts were present only in patient B. It remains unclear whether patient A would require more time to present evidence of immune restoration or belongs to the group of immunological non-responders (INRs). The term INRs is used to describe patients who fail to restore their CD4 lymphocyte count despite receiving optimal treatment and suppression of viral replication. Such impaired response of the immune system is linked to an increased risk of disease progression and death (Yang et al. 2020). Since no consensus on the exact criteria of INRs has been reached and the patient died, we can only surmise that he indeed was an INR.

Another notable difference between the discussed patients is the number of JCPyV DNA copies/ml in the CSF on admission. A significantly greater number was detected in patient A compared to patient B (113,000,000 copies/ml vs. 396 copies/ml), yet the disease progressed very similarly in both cases. According to a study by Delbue et al. (2012), the JCPyV load in the CSF determined at the time of the diagnosis may be indicative of the prognosis. However, we found no confirmation for this observation in our patients.

## Conclusions

Patients with neurological symptoms must be a matter of great concern to all doctors. They should be tested for HIV and examined with an MRI of the brain paired with CSF evaluation earlier even with slight clinical lesions. Treatment that targets JCPyV directly is needed and would be highly beneficial for patients with PML, regardless of the state of their immune system.

## Data Availability

The full data of each case is available upon request.
